# Crystal structure of chlorido­{5,10,15,20-tetra­kis­[2-(2,2-di­methyl­propanamido)­phen­yl]porphyrinato-κ^4^ *N*}iron(III)

**DOI:** 10.1107/S205698901500153X

**Published:** 2015-01-31

**Authors:** Dennis Awasabisah, Douglas R. Powell, George B. Richter-Addo

**Affiliations:** a Department of Chemistry and Biochemistry, University of Oklahoma, 101 Stephenson Pkwy, Norman, OK 73019, USA

**Keywords:** crystal structure, picket-fence porphyrin, C—H⋯O inter­actions

## Abstract

The title compound, [Fe(C_64_H_64_N_8_O_4_)Cl], is a five-coordinate square-pyramidal porphyrin complex with a chloride ion in the axial position, being coordinated from the protected side of the porphyrin; the Fe^III^ atom is displaced by 0.474 (5) Å from the 24-atom mean plane of the porphyrin core towards the chloride. The porphyrin moiety is a ‘picket-fence’ 5,10,15,20-tetra­kis­[2-(2,2-di­methyl­propanamido)­phen­yl]porph­yrinate (por) group. The Fe—Cl bond length is 2.221 (2) Å and the Fe—N(por) bond lengths are in the range 2.043 (5)–2.063 (5) Å. The supra­molecular architecture of the crystal is sustained by C—H⋯O inter­actions between the pyrrolic and phenyl H atoms of one mol­ecule and the carbonyl O atoms of the 2,2-di­methyl­propanamido groups of adjacent mol­ecules. The methyl groups of three of the four *tert*-butyl substituents exhibited rotational disorder over two positions. The investigated crystal was twinned by a twofold rotation about the (001) axis with a refined twin ratio of 0.4086 (16).

## Related literature   

For the synthesis of (T_piv_PP)FeCl (piv = *ortho*-pivalamido), see: Collman *et al.* (1975 ▸). For the crystal structures of other neutral and anionic (T_piv_PP)FeCl complexes, see: Dhifet *et al.* (2011 ▸); Schappacher *et al.* (1983 ▸). For related synthetic applications of the title compound, see: Cheng *et al.* (2000 ▸); Nasri *et al.* (1997 ▸); Bominaar *et al.* (1992 ▸); Gismelseed *et al.* (1990 ▸).
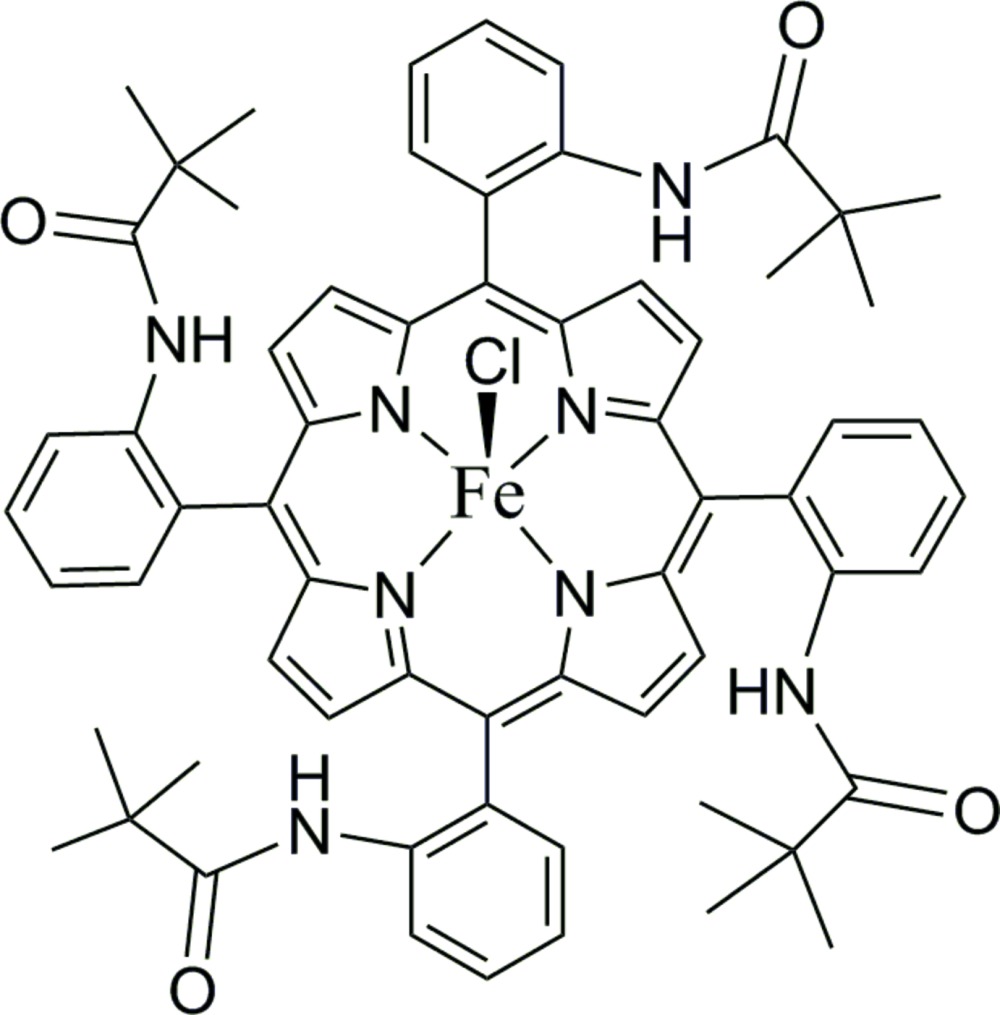


## Experimental   

### Crystal data  

[Fe(C_64_H_64_N_8_O_4_)Cl]
*M* *_r_* = 1100.53
Monoclinic, 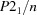

*a* = 17.763 (3) Å
*b* = 17.652 (3) Å
*c* = 20.145 (4) Å
β = 110.570 (4)°
*V* = 5913.8 (18) Å^3^
*Z* = 4
Mo *K*α radiation
μ = 0.35 mm^−1^
*T* = 100 K
0.41 × 0.24 × 0.11 mm

### Data collection  

Bruker APEX CCD diffractometer
Absorption correction: multi-scan (*SADABS*; Bruker, 2002 ▸) *T*_min_ = 0.869, *T*_max_ = 0.962
69995 measured reflections
18970 independent reflections
9442 reflections with *I* > 2σ(*I*)
*R*_int_ = 0.077

### Refinement  

*R*[*F* ^2^ > 2σ(*F* ^2^)] = 0.092
*wR*(*F* ^2^) = 0.280
*S* = 1.01
18970 reflections
788 parameters
279 restraints
H-atom parameters constrained
Δρ_max_ = 1.25 e Å^−3^
Δρ_min_ = −0.45 e Å^−3^

### 

Data collection: *SMART* (Bruker, 2007 ▸); cell refinement: *SAINT* (Bruker, 2007 ▸); data reduction: *SAINT*; program(s) used to solve structure: *SHELXS97* (Sheldrick, 2008 ▸); program(s) used to refine structure: *SHELXL2014* (Sheldrick, 2015 ▸); molecular graphics: *XP* in *SHELXTL* (Sheldrick, 2008 ▸); software used to prepare material for publication: *SHELXL2014*.

## Supplementary Material

Crystal structure: contains datablock(s) I, New_Global_Publ_Block. DOI: 10.1107/S205698901500153X/tk5357sup1.cif

Structure factors: contains datablock(s) I. DOI: 10.1107/S205698901500153X/tk5357Isup2.hkl

**Fig. 1.**
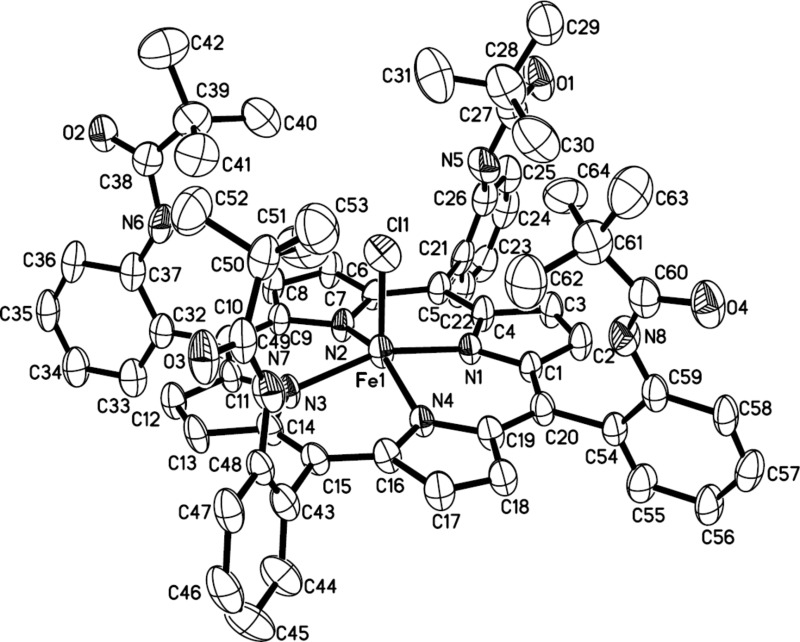
The molecular structure of the title compound (I), showing the atom-labelling scheme and displacement ellipsoids drawn at the 50% probability level. Disordered groups and H atoms have been omitted for clarity.

. DOI: 10.1107/S205698901500153X/tk5357fig1.tif
The mol­ecular structure of the title compound (I), showing the atom-labelling scheme and displacement ellipsoids drawn at the 50% probability level. Disordered groups and H atoms have been omitted for clarity.

**Fig. 2.**
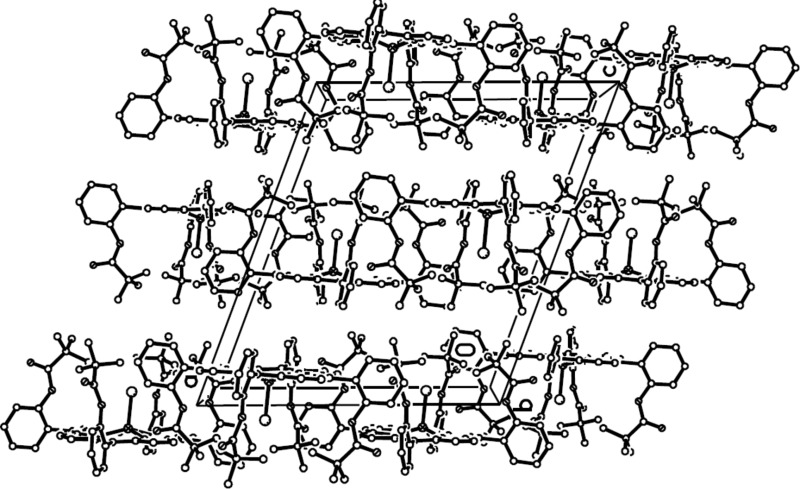
The packing arrangement of molecules of the title compound (I).

. DOI: 10.1107/S205698901500153X/tk5357fig2.tif
The packing arrangement of mol­ecules of the title compound (I).

CCDC reference: 1045127

Additional supporting information:  crystallographic information; 3D view; checkCIF report

## Figures and Tables

**Table 1:** Hydrogen-bond geometry (, )

*D*H*A*	*D*H	H*A*	*D* *A*	*D*H*A*
C2H2O1^i^	0.95	2.43	3.310(8)	154
C7H7O2^ii^	0.95	2.35	3.228(8)	154
C12H12O3^iii^	0.95	2.30	3.223(8)	163
C17H17O4^iv^	0.95	2.32	3.251(8)	168
C25H25O1	0.95	2.29	2.884(12)	120
C36H36O2	0.95	2.34	2.932(11)	120
C47H47O3	0.95	2.24	2.847(11)	121
C58H58O4	0.95	2.32	2.909(11)	119

Symmetry codes: (i) 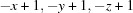; (ii) 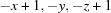; (iii) 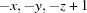; (iv) 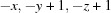.

## supplementary crystallographic information

### S1. Comment

The iron(III) porphyrin complex (T_piv_PP)FeCl has been used as a precursor for the preparation of several (T_piv_PP)Fe derivatives in our laboratory (Cheng *et al.*, 2000) and those of several research groups (Nasri *et al.*, 1997; Bominaar *et al.*, 1992; Gismelseed *et al.*, 1990). The molecular structure of the title compound is shown in Fig. 1. The complex (T_piv_PP)FeCl is square pyramidal and has the chloride atom bonded to iron at the axial position. The chloride ion is coordinated at the protecting 2,2-dimethylpropanamido side of the porphyrin, in contrast to a similar structure reported (Dhifet *et al.*, 2011) where the chloride ligand was on the opposite side of the protecting group. The Fe–Cl bond length is 2.221 (2) Å and the Fe–N(por) bond lengths are in the 2.043 (5)-2.063 (5) Å range. The iron(III) atom is displaced by 0.474 (5) Å from the 24-atom mean plane of the porphyrin core. An Fe–Cl bond length of 2.301 (2) Å and a mean Fe–N(por) distance of 2.108 (±0.015) Å were observed in the related anionic [(T_piv_PP)FeCl]^–^ compound (Schappacher *et al.* 1983). Similarly, Dhifet and coworkers (Dhifet *et al.*, 2011) determined an Fe–N(por) distance of 2.065 (2) Å, an Fe–Cl distance of 2.207 (2) and an iron displacement of 0.420 (4) Å from the 24 atom mean plane of the porphyrin macrocycle.

### S2. Experimental

The (T_piv_PP)FeCl complex with the chloride ion coordinated to iron at the protected side of the porphyrin was obtained serendipitously as follows: To a Schlenk tube equipped with a magnetic stirrer was added (T_piv_PP)FeCl (Collman *et al.*, 1975) (50 mg, 0.045 mmol) and toluene (10 mL). *p*-Fluorophenylmagnesium bromide in THF (0.05 mL, 0.05 mmol) was then added drop-wise and the mixture stirred under N_2_ in the dark for 24 h. The resulting red solution was filtered into a clean Schlenk tube and the toluene solution reduced to ca 3 mL under vacuum. Hexane (10 mL) was added to the solution and placed in a -20 °C freezer overnight. The solid obtained was collected by filtration and dried in vacuo to give a black microcrystalline product. The IR (KBr) spectrum of the product shows a strong ν_CO_ band at 1694 cm^-1^. X-ray quality crystals were obtained from slow evaporation of dichloromethane/ hexane solution of the complex at room temperature under N_2_. We are unsure if the title complex with the chloride in the protected porphyrin cavity was present as a component of our bulk starting reagent (T_piv_PP)FeCl.

### S3. Refinement

H atoms were located geometrically and refined using a riding model on their parent atoms, with C—H = 0.95 Å for aromatic and 0.98 Å for aliphatic, with *U*_iso_(H) = 1.2–1.5*U*_eq_(C). The selected crystal was twinned by a 2-fold rotation about the (0 0 1) axis with a refined twin ratio of 0.4086 (16). The methyl groups of three of the *t*-butyl substituents were disordered: the occupancies of atoms C29 – C31 were refined to 0.759 (13) and 0.241 (13) for the unprimed and primed atoms; the occupancies of atoms C40 – C42 refined to 0.894 (9) and 0.106 (9) for the unprimed and primed atoms; and the occupancies for atoms C62 – C64 refined to 0.807 (9) and 0.192 (9) for the unprimed and primed atoms. The 1–2 and 1–3 distances of the carbons of the disordered methyl groups were set to approximately equal. The displacement parameters of the disordered carbons were restrained to be approximately equal along bonds. Two reflections, *i.e*. (-4 1 1) and (-5 6 4), were omitted from the final refinement owing to poor agreement. A single large (1.25 e Å^3^) peak was observed in the difference map about half way between the Fe and Cl

### Crystal data

**Table:**

[Fe(C_64_H_64_N_8_O_4_)Cl]	*F*(000) = 2316
*M**_r_* = 1100.53	*D*_x_ = 1.236 Mg m^−^^3^
Monoclinic, *P*2_1_/*n*	Mo *K*α radiation, λ = 0.71073 Å
*a* = 17.763 (3) Å	Cell parameters from 5492 reflections
*b* = 17.652 (3) Å	θ = 2.2–23.8°
*c* = 20.145 (4) Å	µ = 0.35 mm^−^^1^
β = 110.570 (4)°	*T* = 100 K
*V* = 5913.8 (18) Å^3^	Plate, black
*Z* = 4	0.41 × 0.24 × 0.11 mm

### Data collection

**Table:**

Bruker APEX CCD diffractometer	9442 reflections with *I* > 2σ(*I*)
φ and ω scans	*R*_int_ = 0.077
Absorption correction: multi-scan (*SADABS*; Bruker, 2002)	θ_max_ = 26.0°, θ_min_ = 1.7°
*T*_min_ = 0.869, *T*_max_ = 0.962	*h* = −21→19
69995 measured reflections	*k* = 0→21
18970 independent reflections	*l* = 0→24

### Refinement

**Table:**

Refinement on *F*^2^	Primary atom site location: structure-invariant direct methods
Least-squares matrix: full	Secondary atom site location: difference Fourier map
*R*[*F*^2^ > 2σ(*F*^2^)] = 0.092	Hydrogen site location: inferred from neighbouring sites
*wR*(*F*^2^) = 0.280	H-atom parameters constrained
*S* = 1.01	*w* = 1/[σ^2^(*F*_o_^2^) + (0.150*P*)^2^] where *P* = (*F*_o_^2^ + 2*F*_c_^2^)/3
18970 reflections	(Δ/σ)_max_ = 0.005
788 parameters	Δρ_max_ = 1.25 e Å^−^^3^
279 restraints	Δρ_min_ = −0.45 e Å^−^^3^

### Special details

**Table:**

Geometry. All e.s.d.'s (except the e.s.d. in the dihedral angle between two l.s. planes) are estimated using the full covariance matrix. The cell e.s.d.'s are taken into account individually in the estimation of e.s.d.'s in distances, angles and torsion angles; correlations between e.s.d.'s in cell parameters are only used when they are defined by crystal symmetry. An approximate (isotropic) treatment of cell e.s.d.'s is used for estimating e.s.d.'s involving l.s. planes.
Refinement. Refined as a 2-component twin. Twinned about (0 0 1) with twin ratio of

### Fractional atomic coordinates and isotropic or equivalent isotropic displacement parameters (Å^2^)

**Table:**

	*x*	*y*	*z*	*U*_iso_*/*U*_eq_	Occ. (<1)
Fe1	0.28128 (5)	0.24482 (5)	0.58789 (6)	0.0518 (3)	
Cl1	0.24722 (12)	0.24555 (12)	0.47072 (11)	0.0795 (6)	
O1	0.5890 (4)	0.3629 (3)	0.4317 (4)	0.101 (2)	
O2	0.3379 (3)	−0.1138 (3)	0.4215 (3)	0.0749 (17)	
O3	−0.1459 (3)	0.1483 (3)	0.3801 (3)	0.0772 (17)	
O4	0.1158 (3)	0.6157 (3)	0.4189 (3)	0.0829 (17)	
N1	0.3581 (3)	0.3363 (3)	0.6157 (3)	0.0481 (15)	
N2	0.3815 (3)	0.1779 (3)	0.6188 (3)	0.0492 (15)	
N3	0.2198 (3)	0.1531 (3)	0.6065 (3)	0.0459 (15)	
N4	0.1959 (3)	0.3119 (3)	0.6070 (3)	0.0461 (14)	
N5	0.5293 (4)	0.3116 (4)	0.5038 (5)	0.080 (2)	
H5N	0.4833	0.2926	0.5032	0.096*	
N6	0.3134 (3)	−0.0315 (3)	0.4977 (4)	0.0672 (19)	
H6N	0.2970	0.0153	0.4996	0.081*	
N7	−0.0328 (3)	0.1885 (3)	0.4671 (4)	0.0624 (17)	
H7N	0.0168	0.2031	0.4745	0.075*	
N8	0.1891 (4)	0.5272 (3)	0.4954 (4)	0.0695 (19)	
H8N	0.2090	0.4814	0.4968	0.083*	
C1	0.3360 (4)	0.4114 (3)	0.6154 (4)	0.0470 (18)	
C2	0.4011 (4)	0.4596 (4)	0.6177 (4)	0.063 (2)	
H2	0.4004	0.5135	0.6174	0.076*	
C3	0.4639 (4)	0.4154 (3)	0.6202 (4)	0.065 (2)	
H3	0.5159	0.4317	0.6229	0.078*	
C4	0.4360 (4)	0.3372 (3)	0.6180 (4)	0.059 (2)	
C5	0.4847 (4)	0.2743 (3)	0.6208 (4)	0.054 (2)	
C6	0.4583 (4)	0.1992 (3)	0.6222 (4)	0.0494 (18)	
C7	0.5074 (4)	0.1332 (3)	0.6258 (4)	0.060 (2)	
H7	0.5615	0.1327	0.6273	0.072*	
C8	0.4617 (4)	0.0720 (4)	0.6265 (4)	0.058 (2)	
H8	0.4781	0.0205	0.6296	0.070*	
C9	0.3844 (4)	0.0992 (3)	0.6216 (4)	0.0451 (17)	
C10	0.3208 (4)	0.0526 (3)	0.6185 (4)	0.0504 (19)	
C11	0.2430 (4)	0.0783 (3)	0.6108 (4)	0.0466 (18)	
C12	0.1769 (4)	0.0300 (4)	0.6064 (4)	0.057 (2)	
H12	0.1773	−0.0238	0.6071	0.068*	
C13	0.1142 (4)	0.0750 (3)	0.6010 (4)	0.056 (2)	
H13	0.0623	0.0583	0.5983	0.067*	
C14	0.1383 (4)	0.1522 (4)	0.6002 (4)	0.0510 (19)	
C15	0.0918 (4)	0.2151 (4)	0.5979 (4)	0.0476 (18)	
C16	0.1186 (4)	0.2891 (3)	0.6015 (4)	0.0500 (19)	
C17	0.0720 (4)	0.3562 (4)	0.6020 (4)	0.065 (2)	
H17	0.0172	0.3569	0.5982	0.078*	
C18	0.1189 (4)	0.4168 (4)	0.6086 (4)	0.064 (2)	
H18	0.1040	0.4681	0.6112	0.077*	
C19	0.1968 (4)	0.3895 (3)	0.6112 (4)	0.0523 (19)	
C20	0.2623 (4)	0.4376 (3)	0.6168 (4)	0.0529 (19)	
C21	0.5717 (4)	0.2871 (4)	0.6281 (5)	0.063 (2)	
C22	0.6307 (5)	0.2797 (4)	0.6937 (5)	0.076 (3)	
H22	0.6167	0.2645	0.7331	0.091*	
C23	0.7124 (5)	0.2945 (4)	0.7030 (6)	0.084 (3)	
H23	0.7538	0.2902	0.7481	0.100*	
C24	0.7288 (5)	0.3154 (4)	0.6437 (6)	0.085 (3)	
H24	0.7831	0.3260	0.6491	0.102*	
C25	0.6720 (5)	0.3218 (4)	0.5778 (6)	0.078 (3)	
H25	0.6867	0.3361	0.5386	0.093*	
C26	0.5915 (5)	0.3069 (4)	0.5689 (6)	0.069 (2)	
C27	0.5277 (6)	0.3408 (4)	0.4410 (6)	0.094 (3)	
C28	0.4473 (6)	0.3423 (4)	0.3827 (5)	0.107 (3)	
C29	0.4548 (7)	0.3817 (7)	0.3172 (5)	0.108 (4)	0.759 (13)
H29A	0.4021	0.3827	0.2791	0.162*	0.759 (13)
H29B	0.4739	0.4338	0.3296	0.162*	0.759 (13)
H29C	0.4931	0.3540	0.3013	0.162*	0.759 (13)
C30	0.3895 (6)	0.3901 (8)	0.4075 (6)	0.126 (4)	0.759 (13)
H30A	0.3365	0.3917	0.3699	0.189*	0.759 (13)
H30B	0.3847	0.3673	0.4502	0.189*	0.759 (13)
H30C	0.4106	0.4418	0.4183	0.189*	0.759 (13)
C31	0.4144 (7)	0.2624 (5)	0.3636 (7)	0.135 (4)	0.759 (13)
H31A	0.3619	0.2648	0.3253	0.203*	0.759 (13)
H31B	0.4517	0.2327	0.3478	0.203*	0.759 (13)
H31C	0.4085	0.2382	0.4053	0.203*	0.759 (13)
C29'	0.4340 (13)	0.4169 (10)	0.3415 (14)	0.119 (5)	0.241 (13)
H29D	0.3811	0.4162	0.3037	0.179*	0.241 (13)
H29E	0.4367	0.4592	0.3737	0.179*	0.241 (13)
H29F	0.4758	0.4230	0.3205	0.179*	0.241 (13)
C30'	0.3798 (7)	0.330 (2)	0.4128 (10)	0.125 (4)	0.241 (13)
H30D	0.3276	0.3316	0.3740	0.187*	0.241 (13)
H30E	0.3869	0.2814	0.4369	0.187*	0.241 (13)
H30F	0.3818	0.3710	0.4467	0.187*	0.241 (13)
C31'	0.4430 (13)	0.2764 (14)	0.3310 (12)	0.118 (5)	0.241 (13)
H31D	0.3905	0.2769	0.2926	0.177*	0.241 (13)
H31E	0.4856	0.2823	0.3111	0.177*	0.241 (13)
H31F	0.4500	0.2282	0.3566	0.177*	0.241 (13)
C32	0.3354 (4)	−0.0323 (4)	0.6227 (4)	0.0540 (19)	
C33	0.3485 (4)	−0.0700 (4)	0.6847 (5)	0.067 (2)	
H33	0.3467	−0.0433	0.7251	0.080*	
C34	0.3646 (4)	−0.1474 (4)	0.6894 (5)	0.074 (2)	
H34	0.3747	−0.1732	0.7330	0.089*	
C35	0.3660 (4)	−0.1858 (4)	0.6316 (5)	0.071 (3)	
H35	0.3779	−0.2385	0.6354	0.085*	
C36	0.3503 (4)	−0.1501 (4)	0.5671 (5)	0.065 (2)	
H36	0.3509	−0.1778	0.5268	0.078*	
C37	0.3334 (4)	−0.0706 (4)	0.5620 (5)	0.059 (2)	
C38	0.3143 (4)	−0.0517 (4)	0.4325 (5)	0.062 (2)	
C39	0.2854 (3)	0.0083 (4)	0.3748 (4)	0.083 (2)	
C40	0.3472 (5)	0.0736 (4)	0.3936 (5)	0.094 (3)	0.894 (9)
H40A	0.3297	0.1129	0.3570	0.140*	0.894 (9)
H40B	0.3514	0.0952	0.4396	0.140*	0.894 (9)
H40C	0.3998	0.0541	0.3962	0.140*	0.894 (9)
C41	0.2019 (5)	0.0379 (5)	0.3702 (5)	0.094 (3)	0.894 (9)
H41A	0.1837	0.0765	0.3330	0.141*	0.894 (9)
H41B	0.1635	−0.0042	0.3589	0.141*	0.894 (9)
H41C	0.2055	0.0601	0.4158	0.141*	0.894 (9)
C42	0.2797 (6)	−0.0275 (5)	0.3032 (4)	0.113 (3)	0.894 (9)
H42A	0.2611	0.0107	0.2656	0.170*	0.894 (9)
H42B	0.3328	−0.0461	0.3062	0.170*	0.894 (9)
H42C	0.2415	−0.0698	0.2923	0.170*	0.894 (9)
C40'	0.3565 (12)	0.033 (2)	0.352 (2)	0.095 (4)	0.106 (9)
H40D	0.3382	0.0712	0.3150	0.143*	0.106 (9)
H40E	0.3996	0.0534	0.3931	0.143*	0.106 (9)
H40F	0.3765	−0.0116	0.3339	0.143*	0.106 (9)
C41'	0.254 (3)	0.0775 (14)	0.4042 (13)	0.090 (5)	0.106 (9)
H41D	0.2353	0.1163	0.3672	0.135*	0.106 (9)
H41E	0.2092	0.0618	0.4189	0.135*	0.106 (9)
H41F	0.2973	0.0982	0.4450	0.135*	0.106 (9)
C42'	0.217 (2)	−0.0251 (14)	0.3105 (13)	0.103 (5)	0.106 (9)
H42D	0.1986	0.0134	0.2732	0.154*	0.106 (9)
H42E	0.2377	−0.0691	0.2923	0.154*	0.106 (9)
H42F	0.1726	−0.0408	0.3252	0.154*	0.106 (9)
C43	0.0049 (4)	0.2034 (4)	0.5921 (5)	0.059 (2)	
C44	−0.0138 (4)	0.2080 (5)	0.6522 (5)	0.077 (3)	
H44	0.0267	0.2193	0.6965	0.093*	
C45	−0.0948 (5)	0.1956 (5)	0.6477 (6)	0.094 (3)	
H45	−0.1087	0.1970	0.6891	0.113*	
C46	−0.1506 (5)	0.1820 (4)	0.5847 (6)	0.078 (3)	
H46	−0.2045	0.1746	0.5820	0.094*	
C47	−0.1336 (4)	0.1781 (3)	0.5224 (5)	0.063 (2)	
H47	−0.1749	0.1682	0.4782	0.075*	
C48	−0.0542 (4)	0.1890 (3)	0.5265 (4)	0.0477 (18)	
C49	−0.0750 (5)	0.1693 (4)	0.4000 (5)	0.065 (2)	
C50	−0.0348 (5)	0.1766 (4)	0.3439 (5)	0.070 (2)	
C51	0.0565 (5)	0.1964 (6)	0.3764 (5)	0.112 (4)	
H51A	0.0847	0.1566	0.4099	0.168*	
H51B	0.0789	0.1999	0.3385	0.168*	
H51C	0.0632	0.2450	0.4014	0.168*	
C52	−0.0396 (6)	0.1008 (5)	0.3067 (5)	0.114 (4)	
H52A	−0.0123	0.0620	0.3417	0.171*	
H52B	−0.0961	0.0866	0.2832	0.171*	
H52C	−0.0135	0.1049	0.2714	0.171*	
C53	−0.0760 (6)	0.2391 (5)	0.2927 (5)	0.104 (3)	
H53A	−0.0716	0.2868	0.3187	0.155*	
H53B	−0.0503	0.2446	0.2571	0.155*	
H53C	−0.1329	0.2263	0.2690	0.155*	
C54	0.2511 (4)	0.5210 (4)	0.6223 (5)	0.0521 (19)	
C55	0.2753 (4)	0.5551 (4)	0.6882 (5)	0.071 (2)	
H55	0.2998	0.5253	0.7294	0.085*	
C56	0.2645 (4)	0.6327 (4)	0.6953 (5)	0.073 (2)	
H56	0.2813	0.6561	0.7407	0.088*	
C57	0.2291 (4)	0.6736 (4)	0.6352 (6)	0.073 (3)	
H57	0.2223	0.7265	0.6397	0.088*	
C58	0.2028 (4)	0.6427 (4)	0.5688 (5)	0.064 (2)	
H58	0.1772	0.6733	0.5284	0.077*	
C59	0.2144 (4)	0.5641 (4)	0.5614 (5)	0.059 (2)	
C60	0.1412 (4)	0.5483 (4)	0.4319 (5)	0.070 (2)	
C61	0.1164 (4)	0.4912 (4)	0.3733 (5)	0.085 (2)	
C62	0.0623 (6)	0.4316 (5)	0.3889 (6)	0.110 (3)	0.807 (9)
H62A	0.0460	0.3942	0.3505	0.166*	0.807 (9)
H62B	0.0918	0.4063	0.4338	0.166*	0.807 (9)
H62C	0.0144	0.4563	0.3923	0.166*	0.807 (9)
C63	0.0711 (7)	0.5290 (5)	0.3027 (4)	0.107 (3)	0.807 (9)
H63A	0.0554	0.4907	0.2651	0.160*	0.807 (9)
H63B	0.0228	0.5538	0.3053	0.160*	0.807 (9)
H63C	0.1057	0.5669	0.2923	0.160*	0.807 (9)
C64	0.1900 (5)	0.4507 (5)	0.3677 (5)	0.092 (3)	0.807 (9)
H64A	0.1727	0.4136	0.3292	0.138*	0.807 (9)
H64B	0.2258	0.4878	0.3580	0.138*	0.807 (9)
H64C	0.2187	0.4247	0.4125	0.138*	0.807 (9)
C62'	0.0261 (6)	0.4756 (18)	0.3494 (15)	0.104 (4)	0.193 (9)
H62D	0.0111	0.4382	0.3111	0.155*	0.193 (9)
H62E	0.0129	0.4560	0.3895	0.155*	0.193 (9)
H62F	−0.0036	0.5228	0.3324	0.155*	0.193 (9)
C63'	0.134 (2)	0.5210 (12)	0.3090 (9)	0.101 (4)	0.193 (9)
H63D	0.1177	0.4831	0.2710	0.151*	0.193 (9)
H63E	0.1043	0.5680	0.2923	0.151*	0.193 (9)
H63F	0.1919	0.5308	0.3225	0.151*	0.193 (9)
C64'	0.1605 (18)	0.4161 (8)	0.3960 (10)	0.099 (5)	0.193 (9)
H64D	0.1429	0.3802	0.3563	0.149*	0.193 (9)
H64E	0.2186	0.4245	0.4098	0.149*	0.193 (9)
H64F	0.1485	0.3954	0.4363	0.149*	0.193 (9)

### Atomic displacement parameters (Å^2^)

**Table:**

	*U*^11^	*U*^22^	*U*^33^	*U*^12^	*U*^13^	*U*^23^
Fe1	0.0358 (6)	0.0378 (5)	0.0886 (8)	0.0041 (4)	0.0304 (6)	0.0061 (6)
Cl1	0.0761 (15)	0.0817 (14)	0.0813 (15)	−0.0009 (11)	0.0281 (12)	0.0111 (13)
O1	0.083 (4)	0.071 (4)	0.176 (7)	0.002 (3)	0.077 (5)	0.028 (4)
O2	0.070 (3)	0.048 (3)	0.136 (5)	0.005 (3)	0.072 (4)	−0.006 (3)
O3	0.047 (3)	0.047 (3)	0.127 (5)	−0.008 (2)	0.017 (3)	0.005 (3)
O4	0.062 (4)	0.060 (3)	0.132 (5)	0.012 (3)	0.042 (4)	0.021 (3)
N1	0.036 (3)	0.032 (3)	0.086 (5)	0.004 (2)	0.034 (3)	0.004 (3)
N2	0.033 (3)	0.032 (3)	0.084 (5)	0.002 (2)	0.023 (3)	−0.002 (3)
N3	0.031 (3)	0.028 (3)	0.081 (4)	0.003 (2)	0.023 (3)	0.009 (3)
N4	0.034 (3)	0.034 (3)	0.079 (4)	0.003 (2)	0.031 (3)	0.006 (3)
N5	0.060 (5)	0.076 (5)	0.131 (7)	−0.007 (4)	0.067 (5)	0.007 (5)
N6	0.062 (4)	0.035 (3)	0.104 (6)	0.010 (3)	0.028 (4)	−0.005 (4)
N7	0.036 (4)	0.061 (4)	0.088 (5)	−0.013 (3)	0.018 (4)	0.005 (4)
N8	0.069 (5)	0.048 (4)	0.090 (6)	0.027 (3)	0.027 (4)	0.021 (4)
C1	0.037 (4)	0.030 (3)	0.085 (6)	0.009 (3)	0.035 (4)	0.007 (3)
C2	0.044 (4)	0.033 (4)	0.126 (7)	0.007 (3)	0.045 (5)	0.005 (4)
C3	0.046 (4)	0.039 (4)	0.125 (7)	−0.007 (3)	0.050 (5)	−0.004 (4)
C4	0.041 (4)	0.033 (4)	0.115 (7)	0.003 (3)	0.041 (4)	0.000 (4)
C5	0.041 (4)	0.034 (3)	0.096 (6)	0.005 (3)	0.035 (4)	−0.002 (4)
C6	0.035 (4)	0.038 (4)	0.080 (6)	0.006 (3)	0.026 (4)	−0.005 (4)
C7	0.036 (4)	0.035 (4)	0.111 (7)	0.004 (3)	0.028 (4)	−0.009 (4)
C8	0.043 (4)	0.030 (4)	0.098 (6)	0.001 (3)	0.021 (4)	−0.009 (4)
C9	0.027 (3)	0.033 (3)	0.074 (5)	−0.002 (3)	0.016 (3)	−0.003 (3)
C10	0.033 (4)	0.037 (4)	0.077 (5)	0.008 (3)	0.015 (4)	−0.002 (4)
C11	0.034 (4)	0.033 (3)	0.076 (5)	−0.003 (3)	0.023 (4)	0.006 (3)
C12	0.040 (4)	0.038 (4)	0.098 (6)	0.002 (3)	0.030 (4)	0.008 (4)
C13	0.030 (4)	0.041 (4)	0.101 (6)	−0.004 (3)	0.029 (4)	0.015 (4)
C14	0.035 (4)	0.043 (4)	0.079 (6)	−0.002 (3)	0.025 (4)	0.009 (4)
C15	0.030 (4)	0.046 (4)	0.071 (5)	0.001 (3)	0.023 (4)	0.010 (3)
C16	0.030 (4)	0.040 (4)	0.085 (6)	0.015 (3)	0.026 (4)	0.017 (4)
C17	0.041 (4)	0.048 (4)	0.119 (7)	0.008 (3)	0.044 (5)	0.008 (4)
C18	0.051 (5)	0.042 (4)	0.117 (7)	0.011 (3)	0.053 (5)	0.012 (4)
C19	0.032 (4)	0.040 (4)	0.092 (6)	0.008 (3)	0.032 (4)	0.013 (4)
C20	0.044 (4)	0.035 (4)	0.085 (6)	0.006 (3)	0.030 (4)	0.010 (4)
C21	0.044 (5)	0.033 (4)	0.117 (8)	0.009 (3)	0.034 (5)	−0.015 (4)
C22	0.051 (5)	0.053 (4)	0.125 (8)	0.007 (4)	0.034 (6)	−0.015 (5)
C23	0.052 (5)	0.062 (5)	0.151 (10)	−0.013 (4)	0.054 (6)	−0.032 (6)
C24	0.044 (5)	0.053 (5)	0.169 (11)	0.001 (4)	0.050 (7)	−0.022 (6)
C25	0.043 (5)	0.062 (5)	0.147 (9)	−0.004 (4)	0.057 (6)	−0.006 (5)
C26	0.047 (5)	0.044 (4)	0.131 (9)	0.004 (4)	0.049 (6)	−0.010 (5)
C27	0.088 (8)	0.064 (5)	0.164 (11)	0.018 (5)	0.085 (8)	0.022 (6)
C28	0.082 (7)	0.102 (6)	0.145 (9)	0.012 (5)	0.051 (7)	0.027 (5)
C29	0.112 (8)	0.086 (7)	0.140 (8)	−0.003 (6)	0.060 (6)	0.006 (6)
C30	0.097 (7)	0.140 (8)	0.159 (9)	0.028 (6)	0.068 (6)	0.048 (7)
C31	0.107 (8)	0.113 (6)	0.171 (10)	−0.022 (6)	0.029 (7)	0.033 (6)
C29'	0.111 (9)	0.109 (7)	0.151 (10)	0.013 (8)	0.063 (8)	0.032 (7)
C30'	0.092 (9)	0.129 (10)	0.164 (10)	0.009 (8)	0.058 (7)	0.045 (8)
C31'	0.103 (9)	0.103 (8)	0.151 (10)	−0.015 (8)	0.048 (8)	0.019 (7)
C32	0.032 (4)	0.043 (4)	0.083 (6)	−0.004 (3)	0.016 (4)	−0.004 (4)
C33	0.048 (5)	0.051 (4)	0.092 (7)	0.003 (3)	0.014 (4)	0.008 (4)
C34	0.063 (5)	0.044 (4)	0.110 (8)	0.008 (4)	0.023 (5)	0.011 (5)
C35	0.050 (5)	0.031 (4)	0.129 (8)	0.005 (3)	0.027 (5)	0.003 (5)
C36	0.029 (4)	0.036 (4)	0.123 (8)	0.003 (3)	0.018 (4)	−0.012 (4)
C37	0.029 (4)	0.041 (4)	0.103 (7)	0.004 (3)	0.018 (4)	−0.003 (5)
C38	0.040 (4)	0.060 (5)	0.098 (7)	−0.001 (4)	0.038 (5)	−0.012 (5)
C39	0.078 (5)	0.080 (5)	0.105 (6)	0.024 (4)	0.048 (5)	0.005 (5)
C40	0.088 (6)	0.092 (6)	0.113 (7)	0.012 (4)	0.051 (5)	0.028 (5)
C41	0.072 (5)	0.097 (6)	0.112 (7)	0.034 (5)	0.031 (5)	0.010 (5)
C42	0.111 (7)	0.125 (7)	0.102 (6)	0.051 (6)	0.034 (5)	−0.002 (5)
C40'	0.092 (8)	0.107 (9)	0.104 (9)	0.028 (7)	0.056 (7)	0.016 (8)
C41'	0.080 (8)	0.089 (8)	0.111 (9)	0.032 (7)	0.047 (7)	0.011 (7)
C42'	0.088 (8)	0.112 (9)	0.106 (9)	0.034 (8)	0.032 (8)	0.003 (7)
C43	0.051 (5)	0.039 (4)	0.098 (7)	0.016 (3)	0.040 (5)	0.023 (4)
C44	0.042 (5)	0.099 (7)	0.092 (7)	−0.007 (4)	0.025 (5)	0.020 (5)
C45	0.054 (6)	0.133 (8)	0.111 (9)	0.003 (6)	0.049 (6)	0.032 (7)
C46	0.041 (5)	0.073 (6)	0.126 (9)	−0.006 (4)	0.035 (6)	0.024 (6)
C47	0.039 (4)	0.043 (4)	0.109 (7)	0.002 (3)	0.029 (5)	0.014 (4)
C48	0.036 (4)	0.039 (4)	0.073 (6)	0.000 (3)	0.024 (4)	0.010 (4)
C49	0.051 (5)	0.036 (4)	0.108 (8)	−0.003 (4)	0.029 (5)	0.003 (4)
C50	0.057 (5)	0.052 (5)	0.097 (7)	−0.008 (4)	0.021 (5)	−0.013 (5)
C51	0.075 (7)	0.148 (10)	0.135 (9)	−0.003 (6)	0.064 (7)	−0.003 (7)
C52	0.137 (9)	0.073 (6)	0.152 (10)	0.000 (6)	0.077 (8)	−0.022 (6)
C53	0.108 (8)	0.081 (6)	0.131 (9)	0.012 (6)	0.052 (7)	0.018 (6)
C54	0.039 (4)	0.036 (4)	0.092 (6)	0.008 (3)	0.036 (4)	0.007 (4)
C55	0.056 (5)	0.051 (5)	0.109 (8)	0.015 (4)	0.033 (5)	0.005 (5)
C56	0.063 (5)	0.045 (4)	0.115 (7)	0.021 (4)	0.035 (5)	0.012 (5)
C57	0.050 (5)	0.044 (4)	0.135 (9)	0.004 (4)	0.043 (6)	−0.010 (5)
C58	0.050 (5)	0.034 (4)	0.117 (8)	0.011 (3)	0.041 (5)	0.013 (4)
C59	0.045 (4)	0.039 (4)	0.102 (7)	0.002 (3)	0.037 (5)	0.007 (5)
C60	0.054 (5)	0.052 (5)	0.122 (8)	0.001 (4)	0.053 (6)	0.005 (5)
C61	0.066 (5)	0.080 (5)	0.115 (6)	0.015 (4)	0.040 (5)	0.011 (5)
C62	0.087 (6)	0.092 (7)	0.149 (8)	−0.017 (5)	0.037 (6)	−0.004 (6)
C63	0.099 (7)	0.089 (6)	0.122 (7)	0.034 (5)	0.024 (6)	0.006 (5)
C64	0.105 (6)	0.095 (7)	0.093 (7)	0.044 (5)	0.057 (5)	0.016 (5)
C62'	0.078 (6)	0.089 (9)	0.138 (10)	0.004 (7)	0.029 (7)	0.003 (8)
C63'	0.102 (9)	0.098 (9)	0.109 (8)	0.028 (8)	0.045 (7)	0.013 (7)
C64'	0.097 (8)	0.087 (8)	0.119 (9)	0.026 (7)	0.046 (9)	0.009 (7)

### Geometric parameters (Å, º)

**Table:**

Fe1—N2	2.043 (5)	C31'—H31F	0.9800
Fe1—N1	2.060 (5)	C32—C33	1.361 (10)
Fe1—N3	2.060 (5)	C32—C37	1.387 (10)
Fe1—N4	2.063 (5)	C33—C34	1.393 (9)
Fe1—Cl1	2.221 (2)	C33—H33	0.9500
O1—C27	1.231 (9)	C34—C35	1.355 (11)
O2—C38	1.221 (7)	C34—H34	0.9500
O3—C49	1.237 (8)	C35—C36	1.382 (11)
O4—C60	1.267 (8)	C35—H35	0.9500
N1—C4	1.370 (7)	C36—C37	1.431 (9)
N1—C1	1.382 (7)	C36—H36	0.9500
N2—C9	1.391 (7)	C38—C39	1.522 (11)
N2—C6	1.392 (7)	C39—C40	1.544 (6)
N3—C11	1.377 (7)	C39—C41'	1.545 (7)
N3—C14	1.407 (7)	C39—C42'	1.545 (7)
N4—C19	1.372 (7)	C39—C41	1.545 (6)
N4—C16	1.398 (7)	C39—C40'	1.545 (7)
N5—C27	1.358 (10)	C39—C42	1.546 (6)
N5—C26	1.389 (11)	C40—H40A	0.9800
N5—H5N	0.8800	C40—H40B	0.9800
N6—C38	1.366 (9)	C40—H40C	0.9800
N6—C37	1.399 (9)	C41—H41A	0.9800
N6—H6N	0.8800	C41—H41B	0.9800
N7—C49	1.339 (9)	C41—H41C	0.9800
N7—C48	1.377 (9)	C42—H42A	0.9800
N7—H7N	0.8800	C42—H42B	0.9800
N8—C60	1.317 (9)	C42—H42C	0.9800
N8—C59	1.404 (9)	C40'—H40D	0.9800
N8—H8N	0.8800	C40'—H40E	0.9800
C1—C20	1.397 (8)	C40'—H40F	0.9800
C1—C2	1.425 (8)	C41'—H41D	0.9800
C2—C3	1.348 (8)	C41'—H41E	0.9800
C2—H2	0.9500	C41'—H41F	0.9800
C3—C4	1.462 (8)	C42'—H42D	0.9800
C3—H3	0.9500	C42'—H42E	0.9800
C4—C5	1.394 (8)	C42'—H42F	0.9800
C5—C6	1.410 (8)	C43—C44	1.366 (10)
C5—C21	1.518 (9)	C43—C48	1.391 (10)
C6—C7	1.442 (8)	C44—C45	1.427 (10)
C7—C8	1.354 (8)	C44—H44	0.9500
C7—H7	0.9500	C45—C46	1.329 (11)
C8—C9	1.425 (8)	C45—H45	0.9500
C8—H8	0.9500	C46—C47	1.391 (11)
C9—C10	1.380 (8)	C46—H46	0.9500
C10—C11	1.410 (8)	C47—C48	1.395 (9)
C10—C32	1.518 (9)	C47—H47	0.9500
C11—C12	1.428 (8)	C49—C50	1.539 (11)
C12—C13	1.341 (8)	C50—C53	1.512 (10)
C12—H12	0.9500	C50—C52	1.521 (10)
C13—C14	1.431 (8)	C50—C51	1.561 (10)
C13—H13	0.9500	C51—H51A	0.9800
C14—C15	1.375 (8)	C51—H51B	0.9800
C15—C16	1.384 (8)	C51—H51C	0.9800
C15—C43	1.521 (9)	C52—H52A	0.9800
C16—C17	1.447 (8)	C52—H52B	0.9800
C17—C18	1.334 (9)	C52—H52C	0.9800
C17—H17	0.9500	C53—H53A	0.9800
C18—C19	1.448 (8)	C53—H53B	0.9800
C18—H18	0.9500	C53—H53C	0.9800
C19—C20	1.413 (8)	C54—C55	1.381 (10)
C20—C54	1.494 (8)	C54—C59	1.397 (10)
C21—C22	1.374 (11)	C55—C56	1.397 (9)
C21—C26	1.400 (11)	C55—H55	0.9500
C22—C23	1.422 (10)	C56—C57	1.359 (11)
C22—H22	0.9500	C56—H56	0.9500
C23—C24	1.376 (12)	C57—C58	1.366 (11)
C23—H23	0.9500	C57—H57	0.9500
C24—C25	1.360 (12)	C58—C59	1.419 (9)
C24—H24	0.9500	C58—H58	0.9500
C25—C26	1.403 (9)	C60—C61	1.497 (11)
C25—H25	0.9500	C61—C63	1.520 (7)
C27—C28	1.498 (13)	C61—C64'	1.525 (7)
C28—C31	1.524 (7)	C61—C64	1.528 (7)
C28—C29'	1.529 (8)	C61—C62'	1.529 (7)
C28—C30'	1.537 (8)	C61—C62	1.529 (7)
C28—C29	1.537 (7)	C61—C63'	1.530 (7)
C28—C30	1.543 (7)	C62—H62A	0.9800
C28—C31'	1.544 (8)	C62—H62B	0.9800
C29—H29A	0.9800	C62—H62C	0.9800
C29—H29B	0.9800	C63—H63A	0.9800
C29—H29C	0.9800	C63—H63B	0.9800
C30—H30A	0.9800	C63—H63C	0.9800
C30—H30B	0.9800	C64—H64A	0.9800
C30—H30C	0.9800	C64—H64B	0.9800
C31—H31A	0.9800	C64—H64C	0.9800
C31—H31B	0.9800	C62'—H62D	0.9800
C31—H31C	0.9800	C62'—H62E	0.9800
C29'—H29D	0.9800	C62'—H62F	0.9800
C29'—H29E	0.9800	C63'—H63D	0.9800
C29'—H29F	0.9800	C63'—H63E	0.9800
C30'—H30D	0.9800	C63'—H63F	0.9800
C30'—H30E	0.9800	C64'—H64D	0.9800
C30'—H30F	0.9800	C64'—H64E	0.9800
C31'—H31D	0.9800	C64'—H64F	0.9800
C31'—H31E	0.9800		
			
N2—Fe1—N1	87.0 (2)	C34—C35—H35	119.3
N2—Fe1—N3	87.23 (19)	C36—C35—H35	119.3
N1—Fe1—N3	155.4 (2)	C35—C36—C37	118.9 (8)
N2—Fe1—N4	153.4 (2)	C35—C36—H36	120.6
N1—Fe1—N4	87.7 (2)	C37—C36—H36	120.6
N3—Fe1—N4	86.90 (19)	C32—C37—N6	119.7 (6)
N2—Fe1—Cl1	102.12 (17)	C32—C37—C36	118.4 (8)
N1—Fe1—Cl1	101.06 (17)	N6—C37—C36	121.9 (8)
N3—Fe1—Cl1	103.51 (17)	O2—C38—N6	121.9 (8)
N4—Fe1—Cl1	104.53 (17)	O2—C38—C39	122.1 (7)
C4—N1—C1	105.8 (5)	N6—C38—C39	116.0 (6)
C4—N1—Fe1	125.7 (4)	C38—C39—C40	108.2 (5)
C1—N1—Fe1	126.2 (4)	C38—C39—C41'	109.2 (6)
C9—N2—C6	104.3 (5)	C38—C39—C42'	109.2 (7)
C9—N2—Fe1	127.2 (4)	C41'—C39—C42'	109.9 (6)
C6—N2—Fe1	126.4 (4)	C38—C39—C41	109.9 (5)
C11—N3—C14	105.7 (5)	C40—C39—C41	110.3 (5)
C11—N3—Fe1	126.8 (4)	C38—C39—C40'	109.1 (6)
C14—N3—Fe1	125.9 (4)	C41'—C39—C40'	109.7 (6)
C19—N4—C16	106.3 (5)	C42'—C39—C40'	109.7 (6)
C19—N4—Fe1	126.3 (4)	C38—C39—C42	108.8 (5)
C16—N4—Fe1	125.8 (4)	C40—C39—C42	110.0 (5)
C27—N5—C26	130.7 (8)	C41—C39—C42	109.6 (5)
C27—N5—H5N	114.7	C39—C40—H40A	109.5
C26—N5—H5N	114.7	C39—C40—H40B	109.5
C38—N6—C37	132.6 (6)	H40A—C40—H40B	109.5
C38—N6—H6N	113.7	C39—C40—H40C	109.5
C37—N6—H6N	113.7	H40A—C40—H40C	109.5
C49—N7—C48	130.7 (6)	H40B—C40—H40C	109.5
C49—N7—H7N	114.6	C39—C41—H41A	109.5
C48—N7—H7N	114.6	C39—C41—H41B	109.5
C60—N8—C59	132.2 (7)	H41A—C41—H41B	109.5
C60—N8—H8N	113.9	C39—C41—H41C	109.5
C59—N8—H8N	113.9	H41A—C41—H41C	109.5
N1—C1—C20	125.8 (5)	H41B—C41—H41C	109.5
N1—C1—C2	110.3 (5)	C39—C42—H42A	109.5
C20—C1—C2	123.9 (5)	C39—C42—H42B	109.5
C3—C2—C1	107.9 (6)	H42A—C42—H42B	109.5
C3—C2—H2	126.1	C39—C42—H42C	109.5
C1—C2—H2	126.1	H42A—C42—H42C	109.5
C2—C3—C4	106.2 (6)	H42B—C42—H42C	109.5
C2—C3—H3	126.9	C39—C40'—H40D	109.5
C4—C3—H3	126.9	C39—C40'—H40E	109.5
N1—C4—C5	126.6 (6)	H40D—C40'—H40E	109.5
N1—C4—C3	109.8 (5)	C39—C40'—H40F	109.5
C5—C4—C3	123.5 (6)	H40D—C40'—H40F	109.5
C4—C5—C6	122.9 (6)	H40E—C40'—H40F	109.5
C4—C5—C21	118.8 (6)	C39—C41'—H41D	109.5
C6—C5—C21	118.1 (5)	C39—C41'—H41E	109.5
N2—C6—C5	125.4 (5)	H41D—C41'—H41E	109.5
N2—C6—C7	110.4 (5)	C39—C41'—H41F	109.5
C5—C6—C7	124.2 (6)	H41D—C41'—H41F	109.5
C8—C7—C6	106.9 (6)	H41E—C41'—H41F	109.5
C8—C7—H7	126.6	C39—C42'—H42D	109.5
C6—C7—H7	126.6	C39—C42'—H42E	109.5
C7—C8—C9	107.3 (6)	H42D—C42'—H42E	109.5
C7—C8—H8	126.3	C39—C42'—H42F	109.5
C9—C8—H8	126.3	H42D—C42'—H42F	109.5
C10—C9—N2	125.2 (5)	H42E—C42'—H42F	109.5
C10—C9—C8	123.8 (6)	C44—C43—C48	121.0 (7)
N2—C9—C8	111.0 (5)	C44—C43—C15	118.7 (8)
C9—C10—C11	124.6 (6)	C48—C43—C15	120.3 (7)
C9—C10—C32	117.7 (5)	C43—C44—C45	119.2 (8)
C11—C10—C32	117.7 (5)	C43—C44—H44	120.4
N3—C11—C10	125.1 (5)	C45—C44—H44	120.4
N3—C11—C12	110.3 (5)	C46—C45—C44	119.0 (9)
C10—C11—C12	124.6 (6)	C46—C45—H45	120.5
C13—C12—C11	107.0 (6)	C44—C45—H45	120.5
C13—C12—H12	126.5	C45—C46—C47	123.0 (8)
C11—C12—H12	126.5	C45—C46—H46	118.5
C12—C13—C14	108.7 (6)	C47—C46—H46	118.5
C12—C13—H13	125.6	C46—C47—C48	118.3 (8)
C14—C13—H13	125.6	C46—C47—H47	120.8
C15—C14—N3	125.5 (6)	C48—C47—H47	120.8
C15—C14—C13	126.2 (6)	N7—C48—C43	118.6 (6)
N3—C14—C13	108.2 (5)	N7—C48—C47	121.9 (7)
C14—C15—C16	124.6 (6)	C43—C48—C47	119.4 (7)
C14—C15—C43	118.3 (6)	O3—C49—N7	123.2 (8)
C16—C15—C43	117.0 (5)	O3—C49—C50	118.0 (8)
C15—C16—N4	126.0 (5)	N7—C49—C50	118.8 (7)
C15—C16—C17	125.8 (6)	C53—C50—C52	112.1 (8)
N4—C16—C17	108.2 (5)	C53—C50—C49	108.9 (7)
C18—C17—C16	108.6 (6)	C52—C50—C49	109.1 (7)
C18—C17—H17	125.7	C53—C50—C51	107.8 (7)
C16—C17—H17	125.7	C52—C50—C51	105.9 (7)
C17—C18—C19	106.9 (6)	C49—C50—C51	113.0 (7)
C17—C18—H18	126.5	C50—C51—H51A	109.5
C19—C18—H18	126.5	C50—C51—H51B	109.5
N4—C19—C20	126.5 (5)	H51A—C51—H51B	109.5
N4—C19—C18	109.9 (5)	C50—C51—H51C	109.5
C20—C19—C18	123.6 (6)	H51A—C51—H51C	109.5
C1—C20—C19	123.4 (6)	H51B—C51—H51C	109.5
C1—C20—C54	118.6 (5)	C50—C52—H52A	109.5
C19—C20—C54	118.0 (6)	C50—C52—H52B	109.5
C22—C21—C26	120.6 (8)	H52A—C52—H52B	109.5
C22—C21—C5	119.1 (8)	C50—C52—H52C	109.5
C26—C21—C5	120.4 (8)	H52A—C52—H52C	109.5
C21—C22—C23	120.5 (9)	H52B—C52—H52C	109.5
C21—C22—H22	119.7	C50—C53—H53A	109.5
C23—C22—H22	119.7	C50—C53—H53B	109.5
C24—C23—C22	116.9 (10)	H53A—C53—H53B	109.5
C24—C23—H23	121.6	C50—C53—H53C	109.5
C22—C23—H23	121.6	H53A—C53—H53C	109.5
C25—C24—C23	124.0 (8)	H53B—C53—H53C	109.5
C25—C24—H24	118.0	C55—C54—C59	119.9 (7)
C23—C24—H24	118.0	C55—C54—C20	119.8 (7)
C24—C25—C26	118.9 (9)	C59—C54—C20	120.3 (7)
C24—C25—H25	120.6	C54—C55—C56	121.3 (8)
C26—C25—H25	120.6	C54—C55—H55	119.4
N5—C26—C21	117.7 (7)	C56—C55—H55	119.4
N5—C26—C25	123.1 (9)	C57—C56—C55	117.8 (9)
C21—C26—C25	119.2 (9)	C57—C56—H56	121.1
O1—C27—N5	122.2 (10)	C55—C56—H56	121.1
O1—C27—C28	121.7 (8)	C56—C57—C58	123.5 (7)
N5—C27—C28	116.1 (7)	C56—C57—H57	118.2
C27—C28—C31	111.0 (6)	C58—C57—H57	118.2
C27—C28—C29'	111.3 (7)	C57—C58—C59	118.8 (8)
C27—C28—C30'	110.4 (7)	C57—C58—H58	120.6
C29'—C28—C30'	109.8 (7)	C59—C58—H58	120.6
C27—C28—C29	109.6 (6)	C54—C59—N8	118.4 (6)
C31—C28—C29	110.1 (6)	C54—C59—C58	118.7 (8)
C27—C28—C30	108.3 (6)	N8—C59—C58	122.9 (8)
C31—C28—C30	110.4 (6)	O4—C60—N8	121.9 (8)
C29—C28—C30	107.4 (6)	O4—C60—C61	119.0 (7)
C27—C28—C31'	108.9 (7)	N8—C60—C61	119.2 (6)
C29'—C28—C31'	108.7 (7)	C60—C61—C63	110.7 (6)
C30'—C28—C31'	107.6 (7)	C60—C61—C64'	111.8 (7)
C28—C29—H29A	109.5	C60—C61—C64	110.5 (6)
C28—C29—H29B	109.5	C63—C61—C64	108.9 (6)
H29A—C29—H29B	109.5	C60—C61—C62'	110.7 (7)
C28—C29—H29C	109.5	C64'—C61—C62'	108.1 (7)
H29A—C29—H29C	109.5	C60—C61—C62	109.4 (6)
H29B—C29—H29C	109.5	C63—C61—C62	109.2 (6)
C28—C30—H30A	109.5	C64—C61—C62	108.0 (6)
C28—C30—H30B	109.5	C60—C61—C63'	110.2 (7)
H30A—C30—H30B	109.5	C64'—C61—C63'	108.4 (7)
C28—C30—H30C	109.5	C62'—C61—C63'	107.5 (7)
H30A—C30—H30C	109.5	C61—C62—H62A	109.5
H30B—C30—H30C	109.5	C61—C62—H62B	109.5
C28—C31—H31A	109.5	H62A—C62—H62B	109.5
C28—C31—H31B	109.5	C61—C62—H62C	109.5
H31A—C31—H31B	109.5	H62A—C62—H62C	109.5
C28—C31—H31C	109.5	H62B—C62—H62C	109.5
H31A—C31—H31C	109.5	C61—C63—H63A	109.5
H31B—C31—H31C	109.5	C61—C63—H63B	109.5
C28—C29'—H29D	109.5	H63A—C63—H63B	109.5
C28—C29'—H29E	109.5	C61—C63—H63C	109.5
H29D—C29'—H29E	109.5	H63A—C63—H63C	109.5
C28—C29'—H29F	109.5	H63B—C63—H63C	109.5
H29D—C29'—H29F	109.5	C61—C64—H64A	109.5
H29E—C29'—H29F	109.5	C61—C64—H64B	109.5
C28—C30'—H30D	109.5	H64A—C64—H64B	109.5
C28—C30'—H30E	109.5	C61—C64—H64C	109.5
H30D—C30'—H30E	109.5	H64A—C64—H64C	109.5
C28—C30'—H30F	109.5	H64B—C64—H64C	109.5
H30D—C30'—H30F	109.5	C61—C62'—H62D	109.5
H30E—C30'—H30F	109.5	C61—C62'—H62E	109.5
C28—C31'—H31D	109.5	H62D—C62'—H62E	109.5
C28—C31'—H31E	109.5	C61—C62'—H62F	109.5
H31D—C31'—H31E	109.5	H62D—C62'—H62F	109.5
C28—C31'—H31F	109.5	H62E—C62'—H62F	109.5
H31D—C31'—H31F	109.5	C61—C63'—H63D	109.5
H31E—C31'—H31F	109.5	C61—C63'—H63E	109.5
C33—C32—C37	120.9 (7)	H63D—C63'—H63E	109.5
C33—C32—C10	120.3 (7)	C61—C63'—H63F	109.5
C37—C32—C10	118.8 (7)	H63D—C63'—H63F	109.5
C32—C33—C34	120.5 (8)	H63E—C63'—H63F	109.5
C32—C33—H33	119.8	C61—C64'—H64D	109.5
C34—C33—H33	119.8	C61—C64'—H64E	109.5
C35—C34—C33	119.9 (8)	H64D—C64'—H64E	109.5
C35—C34—H34	120.1	C61—C64'—H64F	109.5
C33—C34—H34	120.1	H64D—C64'—H64F	109.5
C34—C35—C36	121.4 (7)	H64E—C64'—H64F	109.5
			
C4—N1—C1—C20	−176.7 (7)	C26—N5—C27—O1	7.1 (13)
Fe1—N1—C1—C20	20.1 (11)	C26—N5—C27—C28	−174.9 (7)
C4—N1—C1—C2	−0.2 (8)	O1—C27—C28—C31	115.9 (9)
Fe1—N1—C1—C2	−163.3 (5)	N5—C27—C28—C31	−62.0 (9)
N1—C1—C2—C3	−0.7 (9)	O1—C27—C28—C29'	−44.0 (17)
C20—C1—C2—C3	175.9 (8)	N5—C27—C28—C29'	138.0 (16)
C1—C2—C3—C4	1.2 (9)	O1—C27—C28—C30'	−166.3 (17)
C1—N1—C4—C5	178.3 (8)	N5—C27—C28—C30'	15.8 (17)
Fe1—N1—C4—C5	−18.4 (12)	O1—C27—C28—C29	−5.9 (9)
C1—N1—C4—C3	0.9 (9)	N5—C27—C28—C29	176.2 (8)
Fe1—N1—C4—C3	164.2 (5)	O1—C27—C28—C30	−122.7 (9)
C2—C3—C4—N1	−1.4 (9)	N5—C27—C28—C30	59.3 (8)
C2—C3—C4—C5	−178.9 (8)	O1—C27—C28—C31'	75.8 (17)
N1—C4—C5—C6	−0.4 (13)	N5—C27—C28—C31'	−102.2 (16)
C3—C4—C5—C6	176.7 (7)	C9—C10—C32—C33	100.6 (8)
N1—C4—C5—C21	−175.8 (8)	C11—C10—C32—C33	−80.2 (9)
C3—C4—C5—C21	1.3 (12)	C9—C10—C32—C37	−80.7 (8)
C9—N2—C6—C5	179.8 (7)	C11—C10—C32—C37	98.5 (8)
Fe1—N2—C6—C5	15.5 (10)	C37—C32—C33—C34	3.5 (11)
C9—N2—C6—C7	1.1 (8)	C10—C32—C33—C34	−177.8 (6)
Fe1—N2—C6—C7	−163.2 (5)	C32—C33—C34—C35	−1.1 (11)
C4—C5—C6—N2	2.1 (13)	C33—C34—C35—C36	−1.0 (12)
C21—C5—C6—N2	177.5 (7)	C34—C35—C36—C37	0.7 (11)
C4—C5—C6—C7	−179.4 (8)	C33—C32—C37—N6	174.9 (6)
C21—C5—C6—C7	−4.0 (12)	C10—C32—C37—N6	−3.8 (9)
N2—C6—C7—C8	−1.6 (9)	C33—C32—C37—C36	−3.8 (10)
C5—C6—C7—C8	179.7 (7)	C10—C32—C37—C36	177.5 (5)
C6—C7—C8—C9	1.4 (9)	C38—N6—C37—C32	172.5 (7)
C6—N2—C9—C10	−178.7 (7)	C38—N6—C37—C36	−8.9 (11)
Fe1—N2—C9—C10	−14.6 (10)	C35—C36—C37—C32	1.7 (10)
C6—N2—C9—C8	−0.2 (8)	C35—C36—C37—N6	−177.0 (6)
Fe1—N2—C9—C8	163.9 (5)	C37—N6—C38—O2	−2.4 (11)
C7—C8—C9—C10	177.8 (7)	C37—N6—C38—C39	178.6 (6)
C7—C8—C9—N2	−0.8 (9)	O2—C38—C39—C40	−111.1 (7)
N2—C9—C10—C11	1.0 (12)	N6—C38—C39—C40	68.0 (7)
C8—C9—C10—C11	−177.3 (8)	O2—C38—C39—C41'	176 (2)
N2—C9—C10—C32	−179.8 (7)	N6—C38—C39—C41'	−5 (2)
C8—C9—C10—C32	1.9 (11)	O2—C38—C39—C42'	56 (2)
C14—N3—C11—C10	−179.3 (7)	N6—C38—C39—C42'	−125 (2)
Fe1—N3—C11—C10	14.5 (10)	O2—C38—C39—C41	128.4 (7)
C14—N3—C11—C12	0.8 (8)	N6—C38—C39—C41	−52.5 (7)
Fe1—N3—C11—C12	−165.5 (5)	O2—C38—C39—C40'	−64 (2)
C9—C10—C11—N3	−1.1 (12)	N6—C38—C39—C40'	115 (2)
C32—C10—C11—N3	179.8 (7)	O2—C38—C39—C42	8.4 (8)
C9—C10—C11—C12	178.9 (7)	N6—C38—C39—C42	−172.5 (6)
C32—C10—C11—C12	−0.3 (11)	C14—C15—C43—C44	99.1 (9)
N3—C11—C12—C13	−1.4 (9)	C16—C15—C43—C44	−80.1 (9)
C10—C11—C12—C13	178.6 (8)	C14—C15—C43—C48	−81.6 (8)
C11—C12—C13—C14	1.4 (9)	C16—C15—C43—C48	99.2 (8)
C11—N3—C14—C15	176.5 (7)	C48—C43—C44—C45	1.9 (12)
Fe1—N3—C14—C15	−17.1 (10)	C15—C43—C44—C45	−178.7 (7)
C11—N3—C14—C13	0.1 (8)	C43—C44—C45—C46	−1.9 (13)
Fe1—N3—C14—C13	166.5 (5)	C44—C45—C46—C47	1.0 (14)
C12—C13—C14—C15	−177.4 (7)	C45—C46—C47—C48	−0.1 (12)
C12—C13—C14—N3	−1.0 (9)	C49—N7—C48—C43	172.7 (7)
N3—C14—C15—C16	0.1 (12)	C49—N7—C48—C47	−9.4 (11)
C13—C14—C15—C16	175.9 (7)	C44—C43—C48—N7	177.0 (6)
N3—C14—C15—C43	−179.1 (7)	C15—C43—C48—N7	−2.3 (9)
C13—C14—C15—C43	−3.3 (12)	C44—C43—C48—C47	−1.0 (10)
C14—C15—C16—N4	0.8 (12)	C15—C43—C48—C47	179.7 (5)
C43—C15—C16—N4	180.0 (7)	C46—C47—C48—N7	−177.9 (6)
C14—C15—C16—C17	−177.4 (7)	C46—C47—C48—C43	0.1 (10)
C43—C15—C16—C17	1.7 (11)	C48—N7—C49—O3	0.9 (12)
C19—N4—C16—C15	−178.1 (7)	C48—N7—C49—C50	178.4 (6)
Fe1—N4—C16—C15	15.3 (10)	O3—C49—C50—C53	65.4 (8)
C19—N4—C16—C17	0.4 (8)	N7—C49—C50—C53	−112.3 (8)
Fe1—N4—C16—C17	−166.2 (5)	O3—C49—C50—C52	−57.3 (9)
C15—C16—C17—C18	177.6 (8)	N7—C49—C50—C52	125.1 (7)
N4—C16—C17—C18	−0.9 (9)	O3—C49—C50—C51	−174.8 (7)
C16—C17—C18—C19	1.0 (9)	N7—C49—C50—C51	7.5 (10)
C16—N4—C19—C20	−179.4 (7)	C1—C20—C54—C55	−86.1 (9)
Fe1—N4—C19—C20	−12.9 (11)	C19—C20—C54—C55	95.7 (8)
C16—N4—C19—C18	0.2 (8)	C1—C20—C54—C59	95.4 (8)
Fe1—N4—C19—C18	166.7 (5)	C19—C20—C54—C59	−82.8 (8)
C17—C18—C19—N4	−0.8 (9)	C59—C54—C55—C56	−0.5 (11)
C17—C18—C19—C20	178.8 (8)	C20—C54—C55—C56	−179.0 (6)
N1—C1—C20—C19	−7.0 (12)	C54—C55—C56—C57	0.1 (11)
C2—C1—C20—C19	176.9 (7)	C55—C56—C57—C58	0.9 (12)
N1—C1—C20—C54	174.9 (7)	C56—C57—C58—C59	−1.6 (11)
C2—C1—C20—C54	−1.2 (11)	C55—C54—C59—N8	−178.7 (6)
N4—C19—C20—C1	3.1 (12)	C20—C54—C59—N8	−0.2 (9)
C18—C19—C20—C1	−176.4 (8)	C55—C54—C59—C58	−0.2 (10)
N4—C19—C20—C54	−178.7 (7)	C20—C54—C59—C58	178.3 (6)
C18—C19—C20—C54	1.7 (11)	C60—N8—C59—C54	165.6 (7)
C4—C5—C21—C22	100.0 (9)	C60—N8—C59—C58	−12.9 (12)
C6—C5—C21—C22	−75.6 (9)	C57—C58—C59—C54	1.1 (10)
C4—C5—C21—C26	−79.3 (9)	C57—C58—C59—N8	179.6 (6)
C6—C5—C21—C26	105.0 (8)	C59—N8—C60—O4	9.3 (12)
C26—C21—C22—C23	2.1 (10)	C59—N8—C60—C61	−171.3 (6)
C5—C21—C22—C23	−177.2 (6)	O4—C60—C61—C63	7.3 (8)
C21—C22—C23—C24	−0.7 (11)	N8—C60—C61—C63	−172.2 (7)
C22—C23—C24—C25	−0.6 (12)	O4—C60—C61—C64'	172.8 (16)
C23—C24—C25—C26	0.4 (12)	N8—C60—C61—C64'	−6.6 (17)
C27—N5—C26—C21	167.8 (7)	O4—C60—C61—C64	128.0 (7)
C27—N5—C26—C25	−11.4 (13)	N8—C60—C61—C64	−51.4 (8)
C22—C21—C26—N5	178.5 (6)	O4—C60—C61—C62'	−66.6 (16)
C5—C21—C26—N5	−2.2 (10)	N8—C60—C61—C62'	113.9 (17)
C22—C21—C26—C25	−2.3 (10)	O4—C60—C61—C62	−113.1 (7)
C5—C21—C26—C25	177.0 (6)	N8—C60—C61—C62	67.4 (8)
C24—C25—C26—N5	−179.8 (7)	O4—C60—C61—C63'	52.3 (16)
C24—C25—C26—C21	1.1 (11)	N8—C60—C61—C63'	−127.2 (17)

### Hydrogen-bond geometry (Å, º)

**Table:**

*D*—H···*A*	*D*—H	H···*A*	*D*···*A*	*D*—H···*A*
C2—H2···O1^i^	0.95	2.43	3.310 (8)	154
C7—H7···O2^ii^	0.95	2.35	3.228 (8)	154
C12—H12···O3^iii^	0.95	2.30	3.223 (8)	163
C17—H17···O4^iv^	0.95	2.32	3.251 (8)	168
C25—H25···O1	0.95	2.29	2.884 (12)	120
C36—H36···O2	0.95	2.34	2.932 (11)	120
C47—H47···O3	0.95	2.24	2.847 (11)	121
C58—H58···O4	0.95	2.32	2.909 (11)	119

Symmetry codes: (i) −*x*+1, −*y*+1, −*z*+1; (ii) −*x*+1, −*y*, −*z*+1; (iii) −*x*, −*y*, −*z*+1; (iv) −*x*, −*y*+1, −*z*+1.
