# Supplementary figures and images for: Crystal structure of chlorido­{5,10,15,20-tetra­kis­[2-(2,2-di­methyl­propanamido)­phen­yl]porphyrinato-κ4 N}iron(III)
Source: Acta Crystallogr E Crystallogr Commun. 2015 Jan 31;71(Pt 2):m42–3. doi: 10.1107/S205698901500153X (PMC4384593; doi:10.1107/S205698901500153X)

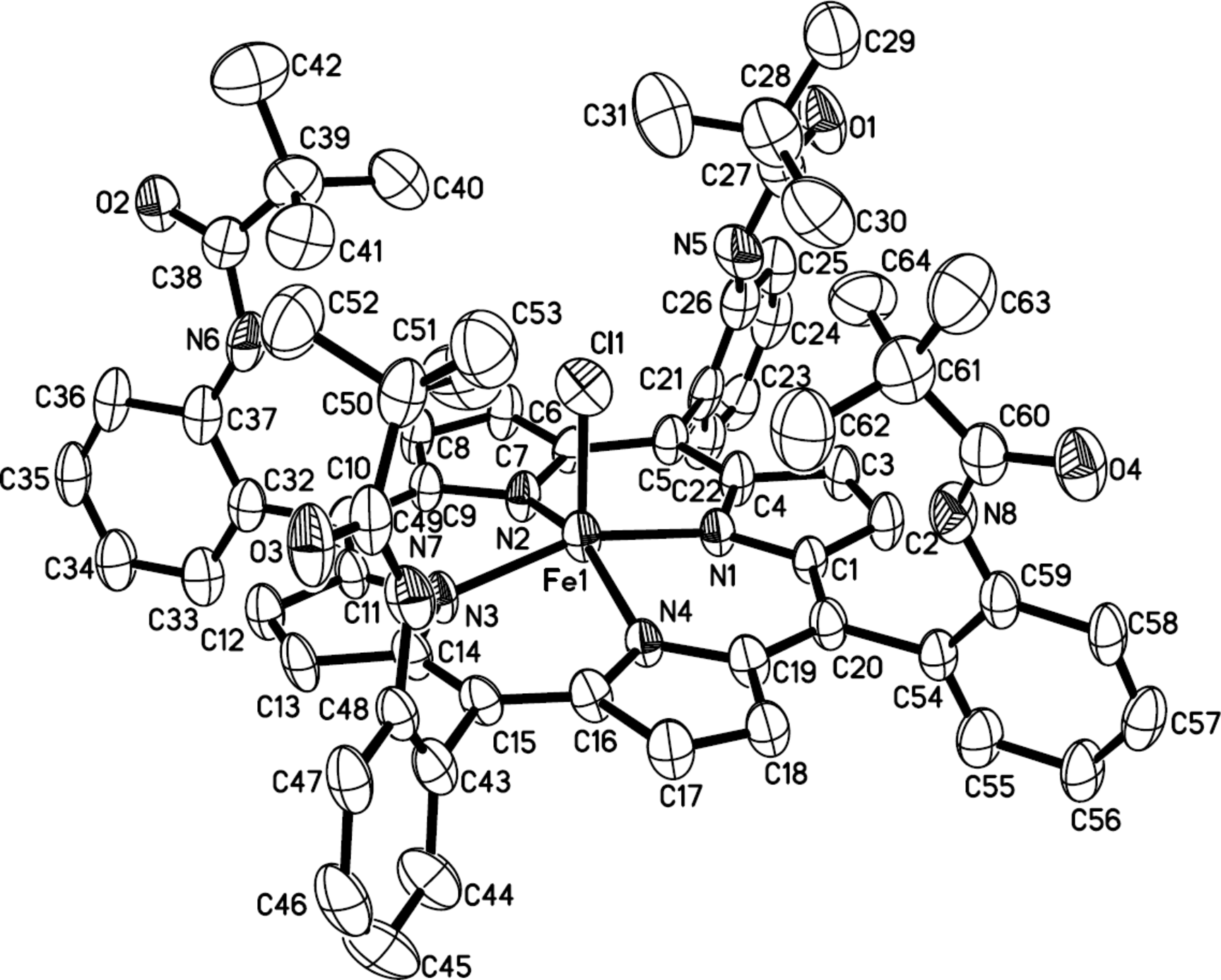

Supplement: Supplementary file 3 [file e-71-00m42-fig1.tif]

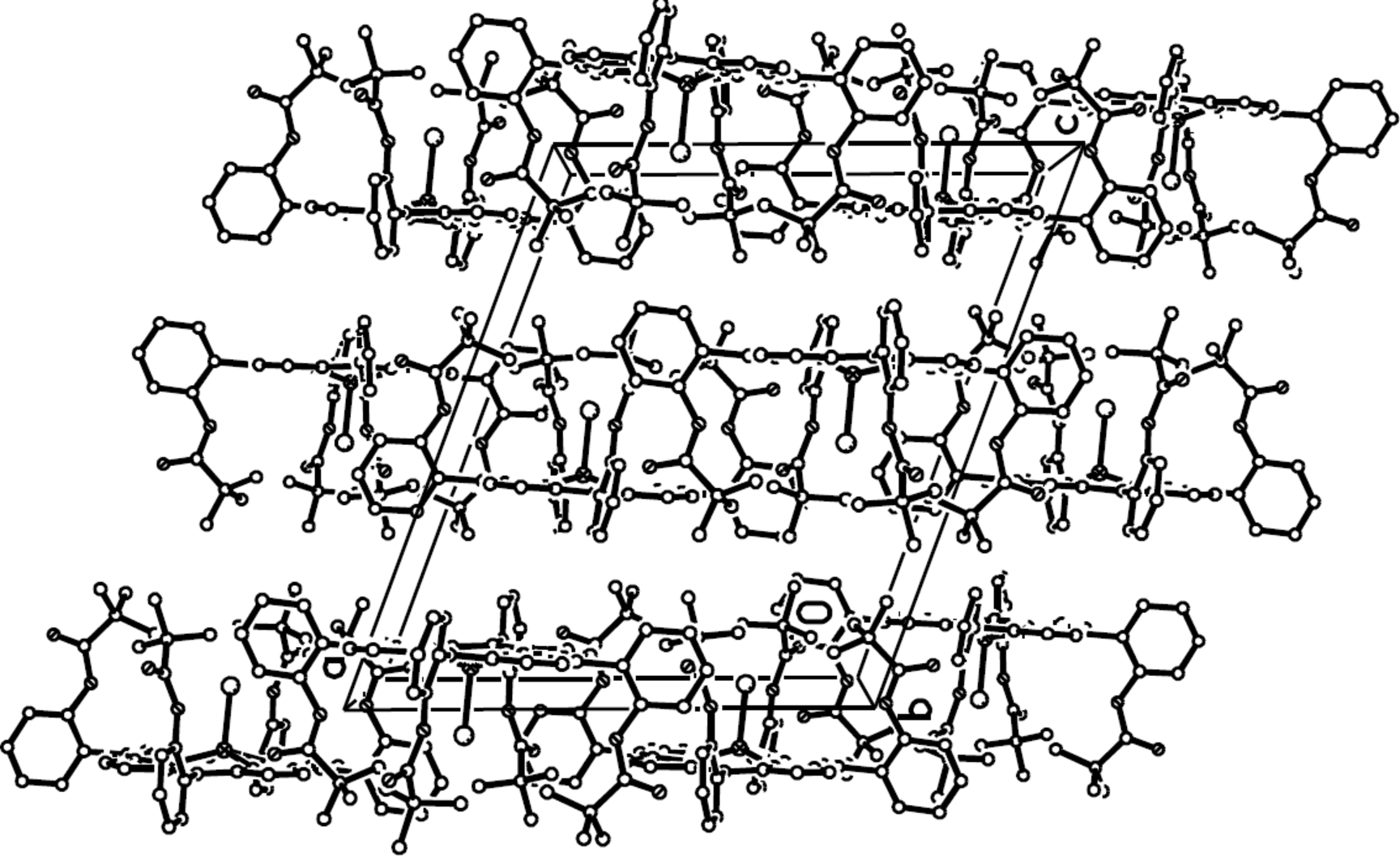

Supplement: Supplementary file 4 [file e-71-00m42-fig2.tif]
